# Multi-Omics Approach in Amelioration of Food Products

**DOI:** 10.3389/fmicb.2022.955683

**Published:** 2022-07-12

**Authors:** Bandita Dutta, Dibyajit Lahiri, Moupriya Nag, Rose Abukhader, Tanmay Sarkar, Siddhartha Pati, Vijay Upadhye, Soumya Pandit, Mohamad Faiz Mohd Amin, Abdel Rahman Mohammad Said Al Tawaha, Manoj Kumar, Rina Rani Ray

**Affiliations:** ^1^Department of Biotechnology, Maulana Abul Kalam Azad University of Technology, Haringhata, India; ^2^Department of Biotechnology, University of Engineering & Management, Kolkata, India; ^3^Faculty of Medicine, Jordan University of Science and Technology, Irbid, Jordan; ^4^Department of Food Processing Technology, Malda Polytechnic, West Bengal State Council of Technical Education, Government of West Bengal, Malda, India; ^5^NatNov Bioscience Private Limited, Balasore, India; ^6^Skills Innovation & Academic Network (SIAN) Institute, Association for Biodiversity Conservation & Research (ABC), Balasore, India; ^7^Center of Research for Development (CR4D), Parul Institute of Applied Sciences (PIAS), Parul University, Vadodara, India; ^8^Department of Life Sciences, Sharda University, Noida, India; ^9^Faculty of Earth Science, Universiti Malaysia Kelantan, Jeli, Malaysia; ^10^Department of Biological Sciences, Al-Hussein Bin Talal University, Ma’an, Jordan; ^11^Chemical and Biochemical Processing Division, ICAR-Central Institute for Research on Cotton Technology, Mumbai, India

**Keywords:** proteomics, transcriptomics, metabolomics, bacteriocin, food products

## Abstract

Determination of the quality of food products is an essential key factor needed for safe-guarding the quality of food for the interest of the consumers, along with the nutritional and sensory improvements that are necessary for delivering better quality products. Bacteriocins are a group of ribosomally synthesized antimicrobial peptides that help in maintaining the quality of food. The implementation of multi-omics approach has been important for the overall enhancement of the quality of the food. This review uses various recent technologies like proteomics, transcriptomics, and metabolomics for the overall enhancement of the quality of food products. The matrix associated with the food products requires the use of sophisticated technologies that help in the extraction of a large amount of information necessary for the amelioration of the food products. This review would provide a wholesome view of how various recent technologies can be used for improving the quality food products and for enhancing their shelf-life.

## Introduction

The last few decades have shown the transition in the outlook on food from being an energy source to an entity that helps in the maintenance of human health. The utility of food has been also extended to a reduction in the risk of diseases (Mtibaa et al., 2019; Ben Braïek et al., 2020). In recent times a visible change has been observed in the development of new food products, production, improvement of various types of packaging materials, and the shelf-life (Capozzi and Bordoni, 2013). The wholesome change in food and food processing has been achieved by the use of various types of current technologies (Prakash et al., 2020). Various types of analytical techniques are being innovated and various types of interdisciplinary methodologies are being implemented for the purpose to develop altered food materials. Bacteriocins have also proved to be effective in food preservation (Ghosh et al., 2021; Kirtonia et al., 2021). The analytical methods that are being used mainly involve the omics approach with the involvement of “high-throughput” technologies. This technology helps in obtaining a large number of measurements in a shorter time-period. The use of “omic” technology helps in the analysis of food and thereby produces a higher analytical quality of food products. This involves genomics, transcriptomics, proteomics, and metabolomic studies that help in and analyzing the food materials and generate data pertaining to genes, proteins, transcripts, and various metabolites (Zheng and Chen, 2014). In recent times, multilevel omic approaches have been used for determining the complexity of biological systems associated with food (Ellis et al., 2018). Thus the utilization of the multi-omics in the field of food sciences is referred to as “foodomics” (Ferranti, 2018). The implications of omic sciences have provided solutions for understanding the implications of various ingredients and food on the human body at a molecular level. The multi-omics approaches at present times are also used for the purpose of detecting various components and metabolites that can be useful for the purpose of human consumption. This review would help in understanding the basis of multi-omics for understanding food quality and also help in the enhancement of food quality. It would also help in bringing a revolution to understand various types of biological structures that constitute food.

## Application of Omics Approach in Food Microbiology

The field of Omic sciences comprises high-throughput sequencing approaches, including transcriptomics and metagenomics along with the use of metabolomics. The field of genomics is used for generating and analyzing the whole genome of the DNA being extracted from the organism (Joyce and Palsson, 2006). Various types of bioinformatics tools can be used for the purpose of analyzing the whole genome of the organism (Herrero et al., 2016). The process of whole-genome sequencing (WGS) acts as a tool for understanding various types of foodborne pathogens. The process of pulsefield gel electrophoresis (PFGE) and multiple-locus variable number tandem repeat analysis (MLVA) are other sub-tools that are being used for determining the food-borne pathogens (Tauxe, 2006; Boxrud et al., 2010). The methods although are new but possess shortcomings in comparison to PFGE and MLVA (Allard et al., 2013). The mechanism of WGS can be used without any discrimination and for a wide number of samples (Achtman, 2012; Barros et al., 2013). It has been observed that techniques like MLVA and PFGE often shows drawback in identifying various subtypes existing within a similar type of pathogen (Zheng et al., 2011; Allard et al., 2012).

## Transcriptomic Analysis of Food Products

A wholesome set of RNA transcripts produced by the genome of an organism that encodes various types of information being present within DNA connecting the phenotype is referred to as a transcriptome. The study of transcriptomics helps in understanding the expression of various types of genes under different types of conditions (Lamas et al., 2019). This study also helps in understanding the various types of functional elements of genome that show various types of molecular interactions with the constituents of the cells (Valdés et al., 2013). This analytical method is used for understanding the expression of the genes which includes both targeted and untargeted analysis. This mechanism is used for the purpose of quantifying some selected transcripts. The targeted mechanism is used for the purpose of quantifying some of the selected transcripts whereas the untargeted mechanism helps to quantitatively analyze the maximum number of gene expressions under a set of transcriptome. This technique involves the use of single-stranded DNA for the purpose of being hybridized with the homologous regions there by forming a stable hydrogen-bonded structure. The mechanism of Microarray works on the same principle in which the probe sequences are casted on the unknown nucleic acid and solid substrate.

The transcriptomic study can be achieved with the involvement of real-time reverse transcription PCR, next-generation RNA sequencing, and microarrays (Lowe et al., 2017). The process of real-time PCR can be used in detecting small changes among the expressions of the genes (Bustin and Nolan, 2004). The evaluation of the transcriptomic samples can be efficiently performed by the use of the microarray technique. Predesigned microarrays can be used for the transcriptomic analysis of various foodborne pathogens like *Escherichia coli* (Lenahan et al., 2014). The use of RNA-seq has become an important reference technique for the performance of the transcriptomic analysis and provides a concrete result in comparison to microarray and RT-PCR techniques (Lamas et al., 2019). At present times, the gold standard method for the process of bacterial subtyping comprises various phenotyping methods, including band-associated molecular methods like pulse-field gel electrophoresis (PFGE) and serotyping. This standard of bacterial identification within the food and food products are complements with omic sciences. The process of pulse field gel electrophoresis and serotyping is used for the purpose of differentiating strains beyond the level of bacterial species and is performed based on the interactions that take place between the antigen and the antibody along with the use of restriction endonucleases (Stenutz et al., 2006). These analyses help in the determining whether the major cause of human listeriosis is associated with *L. monocytogenes* (Maury et al., 2016). The mechanism of serotyping can be used effectively in differentiating between the sub-species. Another recent studies that involves the use of molecular subtyping that mainly helps in the sequencing of specific regions of genes or the entire genome of the organism. The mechanism of multi-locus sequence typing helps in distinguishing between the strains on the variation of gene loci associated with the core genome of the test organism (Feil and Enright, 2004). The nucleotide data obtained by sequencing of the genes help in comparing with the nucleotides found within the database, thus the identification of allelic types can be performed (Feil and Enright, 2004). The process of multilocus sequence typing (MLST) helps in targeting the various regions for the same organism. MLST has been successful in developing L. monocytogenes by targeting various types of housekeeping genes like that of *prfA* virulence gene (Nightingale et al., 2005).

## Genomics Associated With Probiotics

The microorganisms that help in providing health benefit with consumption at adequate proportion are referred to as probiotics (Hill et al., 2014). The probiotic consumed should possess the potential to survive with the gastric transit so that they can provide health benefits to the consumer. The probiotics possess the adhering capacity with the gut mucosa followed by colonization (Bienenstock et al., 2013). It has been observed that the genome of various types of probiotic strains has been sequences resulting in the development of probiogenomics (Lugli et al., 2022). The determination of the action of the probiotics can be studied by the determination of individual genes (Plaza-Diaz et al., 2019). *Lactobacillus* is one of the most crucial organisms that are being used in the development of fermented products. They are the organisms that live in mutualism with the microbiota found within the human gastrointestinal tract. Thus genome sequencing of the organism can help in the study of genes and the mutants associated with the organism (Lebeer et al., 2008). Another study on genome sequencing of *Bifidobacterium breve* revealed that the organism possesses gene clusters that encode type IV tad-pili, which plays a vital role in the cellular adhesion with the host (Alessandri et al., 2021). It has been observed that disruption in the locus of the gene prevents the colonization of the organism within the gut (Alessandri et al., 2021). Studies have revealed by transposon mutagenesis that mane genes are responsible for the colonization of *Lactobacillus casei* within the gut (Palud et al., 2018). Out of all the genes studied, it was found that 47 genes help in the mechanism of colonization within the ileal loop of the rabbit. These genes that are found within the organisms are associated with various types of housekeeping functions comprising the synthesis of cell wall, metabolism of amino acids, synthesis of carbohydrates, and adaptation to environment. Identification of genes responsible for immunomodulation can be observed by the use of functional genomics (Stergiou et al., 2021).

## Proteomics

Proteomics is considered to be another very important part of omic-technology that plays a vital role in dealing with various large-scale proteins that are associated with the biological systems. Various stimuli of the environment are understood by the means of proteome dynamicity and temporal specificity (Pischetsrieder and Baeuerlein, 2009). Proteome is considered to be the set of proteins that are encoded by the genome comprising of various modifications and isoforms along with its interaction with itself and various types of higher-order structures (Tyers and Mann, 2003). The process of mass spectrometry can be used in the characterization and identification of various proteins. The determination of the proteome comprises various steps followed by the analysis using mass spectrometry. The general process of mass spectrometry analysis comprises mass values obtained followed by searching databases like Mascot (Tuli and Ressom, 2009). The isolation of unknown proteins from the food samples can be performed by peptide-mass fingerprinting which is a type of 2D gel electrophoresis. The use of tandem mass spectrometry in peptide fragmentation fingerprinting helps in obtaining data from one or more types of peptides that are associated with the proteins (Eng et al., 1994). The major aim of food proteomics help in the analysis of a particular type of proteome, thereby identifying the potential peptide biomarkers. The process of food proteomics help in searching various types of peptide biomarkers in the various discovery phases of biological samples.

**FIGURE 1:**
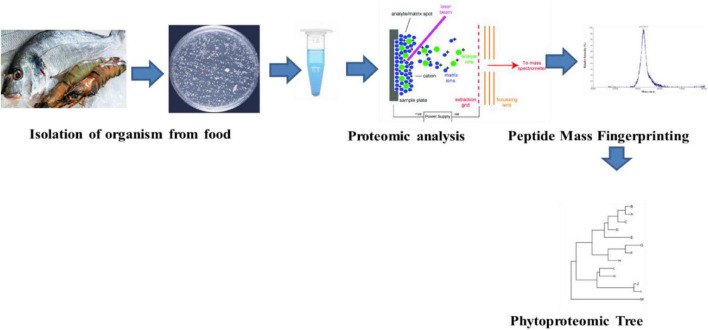
Proteomic analysis of the food associated microorganisms.

Biochemical changes that are brought about within the food products due to the activity of the microbial species cause food spoilage. The extent of the spoilage of food is associated with variation among inherent and non-inherent microflora and associated growth conditions that vary with respect to pH and temperature. The process of identification and classification of the microorganisms is associated with biochemical, morphological, proteomics, and genomics approaches. The technique of MALDI-TOF-MS can be used for analyzing the bacterial cells or the proteins that are responsible for the process of food spoilage (Barbuddhe et al., 2008). Various types of commercial databases have been made on the basis of bacterial identification using MALDI-TOF–MS (Böhme et al., 2012) (Figure 1).

**FIGURE 2:**
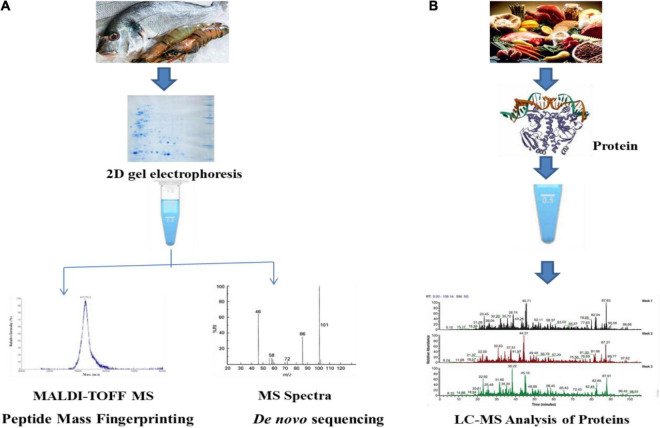
Discovery phase **(A)** and target phase **(B)** analysis of proteomics in food.

The process of the discovery phase helps in assessing a particular type of proteome using various types of reference strains or samples that can act as a potential group of biomarkers. The process of protein identification and quantification can be achieved by the use of bottom-up technique. Proteins like arginine kinase and parvalbumin act as effective biomarkers for the process of identifying shellfish and other fishes (Ortea et al., 2009). The second phase is said to be a target-based approach that helps in monitoring the peptides that are specific to species (Shevchenko et al., 1997). The collection of species-specific peptides is followed by mass spectrometric analysis (Kaur and Anuradha, 2010). The identification of various peptide markers can be obtained by monitoring the transition. High specificity and sensitivity are the two most important advantages of target-based approach (Sauer, 2003) (Figure 2).

## Bacteriocin-Mediated Food Amelioration

Antimicrobial peptides are synthesized by microbial ribosomes, which are named bacteriocins. These bacteriocins are able to eliminate or resist bacterial strains irrespective of the bacterial genus. Bacteriocins are generally effective in the narrow spectrum of bacteria and inhibit a wide assortment of microbes (Cotter et al., 2005). They are considered one of the important natural biopreservatives because they have antimicrobic activity against foodborne microbes. Lactic acid bacteria (LAB) produces bacteriocins that are used worldwide because they particularly function on food fermentation, preservation, and the development of taste or flavor.

Microorganisms are considered pathogens when they are considered infectious carriers causing illness to people, animals, or plants. These pathogens not only affect human well-being but are also effective on food varieties or livestock. Dependence on antibiotics or antimicrobial drugs had opened the road to the expansion of drug-resistant bacteria (Baker et al., 2018). Research on bacteriocin or bacteriocin-like inhibitory substances (BLIS) has pivoted to solve an elective restorative choice for pathogenic diseases. Bacteriocins are the proteinaceous toxin that have proved their potential to replace chemical preservatives in the food industry and antibiotics against several resistant pathogens. Bacteriocins are usually active against other bacterial strains without blemishing the bacteria themselves by specific protective proteins (Riley and Wertz, 2002). The exertion of antibiotics to kill microorganisms interfere with the gut microbiota, by killing the designated microbial group, and also encompasses the microbial group (Langdon et al., 2016). The natural antimicrobial peptide (AMP), i.e., bacteriocin provides a shield to the producing microorganisms from different pathogens or microbes by inhibition and elimination (Yang et al., 2014). Bacteriocins are abundantly found in nature. Around 99% of the bacteria are able to produce bacteriocins among which most of them are unidentified (Riley and Wertz, 2002). LAB, classified as non-sporous and tolerant to acid climate, are considered the main source of bacteriocin production whereas *Bacillus* sp. *Staphylococcus* sp. and *E. coli* are also able to produce bacteriocin (Axelsson, 2004). LAB works significantly in fermentation where it utilizes carbohydrates as the main resource to produce lactic acid as the principal product (Mozzi, 2016). Lactic acid bacteria are found in a few genera, consisting of *Lactococcus, Streptococcus, Alloiococcus, Pediococcus, Leuconostoc, Aerococcus, Carnobacterium, Dolosigranulum, Enterococcus, Oenococcus, Tetragenococcus, Weissella, Vagococcus, and Lactobacillus* (Mokoena, 2017). These are restricted to two categories, among them one is heterofermentative LAB and the other is homofermentative LAB. In the fermentation process carbon dioxide, alcohol, acetic acid, and lactic acid are manufactured by heterofermentative LAB, but heterofermentative LAB produces lactic acid (Oliveira et al., 2017). From earlier research work, it is observed that some LAB were found to have a capability of producing antimicrobial substances, i.e., bacteriocin, for defending themselves against other pathogens and spoilage bacteria. Among bio-preservatives, bacteriocin has grabbed the eye of researchers to be utilized as a characteristic food bio-preservative because of its antimicrobial action against food spoilage by various pathogens. Isolation of various bacteriocin-producing bacteria aims for utilizing bacteriocin as it has applications in food fermentation, flavor development, also in food preservation for human health (Cleveland et al., 2002).

**FIGURE 3:**
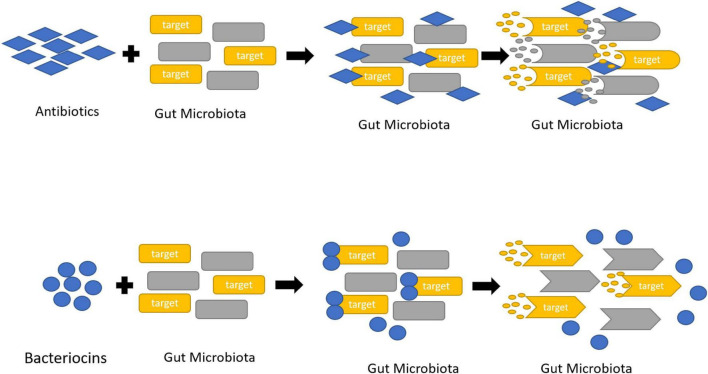
Killing mechanism of antibiotics and bacteriocins against gut microbiota.

The contemporary society has more consternation about food safety as the uses of several chemical food preservatives evoke the toxin effect. Thus uses of natural resources in diets manifest health benefits. Although the natural food additives became more popular compared to the chemical ones, the antibiotics and natural preservatives available in the market are synthesized chemically and they affect the human gut microbiota by reducing their count. Distinctly (GRAS) or the “generally recognized as safe” bacteriocins are valued in the food industry (Figure 3).

## Classification of Bacteriocin

Bacteriocins are mainly classified into three groups, the first group is Class I bacteriocins which are known as lantibiotics, which are small peptides that have the ability to go through post-translational modification to produce active peptides. Bacteriocins of Class I group are made with functional peptides with size less than 5 kDa. It contains different posttranslational transformed deposits like lanthionine and b-methyl lanthionine. Nisin belonging to the class I group is one of the most studied bacteriocins and effective against pathogenic microbes and food spoilage (Maisnier-Patin and Richard, 1996). Bacteriocins of class II group are hydrophobic, firm to heat, and non-modified peptides, which are further divided between the following sub-classes: class IIa and class IIb. Bacteriocins with subclass of Class IIa like pediocin PA1, leucocin A generally falls under food preservation methods because it has pediocin-like Listeria. Between the two complementary peptides found in Class IIb, bacteriocins have synergistic action like enterocin X, and plantaricin A enhances the antimicrobial ramifications. The peptides contain hydrophobic and cationic amphiphilic areas that are effective in nanomolar concentration to picomolar concentrations (Ríos Colombo et al., 2019).Class III bacteriocins are sizeable, heat-sensitive, non-lytic peptides which are vulnerable to bacteriolysis. Class III bacteriocins are composed of enormous proteins whose size exceeds 30 kDa. Enterolysin A, helveticin J, and Lysostaphin are the various examples of class III bacteriocins (Vaičikauskaitė et al., 2019).

## Colicins

Colicins are antimicrobial peptides that are made of three realm:– an amino-terminal translocation domain that has been embroiled in the transmit crossing the outer membrane through the translocator protein; a medial receptor-binding domain particularly attached to the outer membrane receptor of bacteria; and lastly a domain of carboxy-terminal cytotoxic domain possessing properties of antibacterial (Cascales et al., 2007). Colicins are further classified into three types based on their antimicrobial mechanism.

Type I: Pore-forming type colicins: Pores are structured from the inner membrane which leaks the cytoplasmic compounds and loss of iron leads to the cell death.

Type II: Nuclease type colicins: Colicins contain DNase that digest the RNA and DNA of bacteria. An example of colicins is peptidoglycanase, which first digests the peptidoglycans, leading to the incapability to produce peptidoglycan hence causing death of the bacteria (Cascales et al., 2007).

Type III: Immunity proteins with specificity are continuously produced to inactivate colicins for avoiding colicins spike. Colicins can enter the bacterial cells to kill them whereas some non-receptor bacteria are resistant to the colicins. Bacteria with deficient amounts of the translocator protein system are known as tolerant strains, and bacteria with secrete immunity protein are known as immune strains. In several bacteria, colicins are usually encoded by a plasmid; however, some of them are found in chromosomes. Lysis protein, toxin protein, and immunity protein are the three major proteins that are usually encoded by colicin gene clusters. Bacteria, secreting colicin are known to be “bacteriocin release protein” (BRP) and they are usually lysis proteins.

## Microcins

Microcins with low molecular weight ribosomally produced antimicrobial peptides, i.e., hydrophobic in nature, are usually produced as precursor peptides that have high heat tolerance, proteases, and pH. Microcins, which are usually secreted by gram-negative bacteria, are divided into different groups based on their molecular weight, disulfide bonds, and post-translational modifications. Microsins can be distinguished from collision depending upon their molecular weight of 25–80 kDa. The precursor peptides of Microsins include core peptides and N-terminal leader peptides. During the process of maturation for becoming an effective microcin, these precursor peptides sometimes undergo the post-translational modification leading to the maturation of active peptides. *Enterobacteriaceae* secrete microcin, which is highly heat resistant and has high ph. The bactericidal mechanisms of microcins are distinct, which includes formation of pores, nuclease functions like RNase and DNase, and the inhibition of DNA replication or protein synthesis.

i) Class I microcins have low molecular weight peptides.
ii) Class II microcins with large molecular weights are compared to the class I microcins and they do not undergo post translational modifications (Severinov et al., 2007). Class IIa microcin gathers functional peptides through the synthesis process.

## Bacteriocin Obtained From Gram-Positive Bacteria

Bacteriocins synthesized by gram-positive bacteria have similar characteristics to the microcins. Gram-positive bacteria consist of LAB (Lactic Acid Bacteria) which are considered the major producers of bacteriocins including different inhibitory spectrum, physicochemical properties and sizes (Klaenhammer, 1993). Bacteriocins from gram-positive bacteria are assembled into the following groups.

Class I bacteriocins are the modified low molecular weight peptides or lantibiotics with lanthionine (Chatterjee et al., 1992).
Class II bacteriocins are unmodified positively charged peptides without lanthionine.
Class III bacteriocins are heat-labile peptides with high molecular weight. They are further classified into
**Group A bacteriocins-** that causes bacteriolysis and
**Group B bacteriocins-** that are non-lytic proteins (Nilsen et al., 2003).

Antibiotics and food preservatives are usually used to inhibit the microbial growth to prolong the life span of the food. The bacteriocins synthesized from both the gram-positive and gram-negative bacteria can be used as a natural preservative in the food products so that they can easily be digested into human gastrointestinal tract. Hence, bacteriocins are considered to be safe food additives (Cleveland et al., 2001). Lantibiotic Nisin is known to be effective in pervading the biofilm. The increased diffusion properties make them more formidable toward biofilms but also active in a lot of food systems. These derivatives of bacteriocin along with increasing bioactivity can also decrease the costs related with targeting the biofilms (Davison et al., 2010). A study proclaimed that nisin along with the antibiotic ciprofloxacin acted against the Methicillin-resistant *Staphylococcus aureus* (MRSA) biofilms (Dosler and Mataraci, 2013). Bacteriocins usually exhibit pore formation and diffusion of the ATP from biofilm, which is considered to be an important role in the mechanism of action to target MRSA biofilms.

Bacteriocins manifest a heterogenous group considering their molecular weight, stability, physicochemical properties, and antimicrobial spectrum along with their mode of action. Few bacteriocins only have activity against homologous species whereas others have diverse activity mostly against the gram-positive bacteria (De Vuyst and Vandamme, 1992).

At high concentrations, nisin is found as an active agent against gram-negative bacteria where cells are pretreated with EDTA (Stevens et al., 1991). Since nisin is a protein molecule that consists of 34 amino acids with low molecular weight, the synthesis process of nisin is quite complex as it comprises aeration, transcription, transduction, and post-translational modifications. Nisin has two variants, namely nisin A and nisin Z, which are different from one another in only the 27th amino acid, where histidine found in nisin A has been replaced with asparagine in nisinZ. These are generally used in dairy products and canned foods that are highly active to produce cheese spread since processed cheese is impenetrable to heat-liable spore-forming organisms (Deegan et al., 2006). This is active against gram-positive mastitis-causing pathogens. Nisin is also considered as a surfactant as it has cationic nature and cells are treated with nisin causing leakage of material which are UV-absorbing (Jack et al., 1995).

In some cases, it was noted that bacteriocins do not work against gram-negative bacteria. However, the outer lipopolysaccharide layer of gram-negative bacteria acts as a cell barrier that prevents molecules from reaching the cytoplasmic membrane. One such example is *Lactobacillus plantarum, which is* produced in plantaricin 35d, i.e., effective against *Aeromonas hydrophila* (Messi et al., 2001).

The killing mechanism of gram-positive bacteria-mediated bacteriocins involves two processes: the formation of pores and altering the activity of enzymes or quenching sensation. High cationic bacteria like lactinin bind quickly to the membrane of the phospholipid bilayer. The indirect ion channels have been formed due to the interaction between the hydrophobic component of lactinin and the bacterial target membrane and those channels lead to the pore formation in addition to the presence of high trans-membrane energy. The existence of anionic lipids and the truancy of cationic lipids can trigger the outflow of intracellular elements. Type B antimicrobials supress the conversion of enzymes in the target bacteria (Calo-Mata et al., 2008).

Class IIa bacteriocins work by building pores in the cytoplasmic membrane. Bacteria that produce bacteriocin protect themselves from their bacteriocin by antibodies. When these proteins are professed in the sensitive cells, they form a strong defense against bacteriocin. Immune proteins can strongly indicate certain properties in relation to the bacteriocins that give them resistance.

## Bacteriocin-Mediated Applications in Food Industry

All living things, including the land, the sea, the river, and the air, are full of germs. Some of them can cause food and drink contamination by contamination. Contamination and wastage of food and drink are now major problems in the food industry, leading to the damage of the taste of food and beverages and causing foodborne illness in humans (Villalobos-Delgado et al., 2019). The malevolent bacteria causing foodborne infection are divided into five categories, namely, germs, parasites, protozoa, and fungi. Commonly found as drivers that cause foodborne illness and food intoxication, they are called pathogens. Food inebriation is caused by toxins that are induced by micro-organisms in food, which causes a prompt reaction in the human system by the subsequent consumption of contaminated food. One of the examples showed that *Staphylococcus aureus* is responsible for the humans’ digestive tract inflammation after taking enterotoxin-containing foods (Castro et al., 2018). The multiplication of food-borne pathogens in the human digestive system exhibits gastrointestinal infection slowly (Drolia and Bhunia, 2019), for examples, diarrhea in people, where its causative agent is *Salmonella* contamination.

Chemically added materials are utilized broadly for food preservation. However, it causes several human medical issues because of the harmfulness of those added chemicals. It increases the requirement for chemical-free, natural, organic, products utilized for the preservation of food so that it can prevent health issues. Thus, so many studies have been done on bio-additives that can be utilized for restraining microbes to prevent food spoilage (Ben Said et al., 2019). Biopreservation is the process by which non-pathogenic microorganisms or some metabolites are introduced by microorganisms to increase the retention time of food. A very important biosupplements is bacteriocin or BLIS, i.e., enterocin, leucocin, nisin, and pediocin, which has been used in food industry institutions in order to arrest food spoilage (Table 1) (Mei et al., 2019).

**TABLE 1:** Bacteriocins used in the food industry.

Name of the bacteriocin	Organism from which bacteriocin is obtained	Products on which its act	Characteristics	References
BacST202Ch BacST216Ch	*Lactobacillus plantarum*	Pork inhale sausage	SDS, Triton X-100 Tween 20 or Tween 80 along with NaCl, EDTA and urea Low and high temperatures With pH range 2.0–12.0	Todorov et al., 2010
Enterocins	*Enterococcus faecalis* L3B1K3	Ideal raw cheese	Partially purified enterocin in amount of 536 μg/g after 6 h incubation is able to reduce *L. monocytogenes* to not-detectable levels in cheese.	Silva et al., 2018
Enterocins NKR-5-3A, B, C, D, Z	*Enterococcus faecium* NKR-5-3	Fermented fish	Salt, Temperature. *Bacillus/Listeria* *Enterococcus/Lactobacillus*	Perez et al., 2014
Lacticin 3147	*Lactococcus lactis* subsp. *lactis* DPC3147	Cottage cheese, Yogurt	85% of *L. monocytogenes* contamination is being reduced in cottage cheese after 120 min incubation and in yogurt after 10 min incubation to undetectable levels	Morgan et al., 2001
MBSa2 and MBSa3, Sakacin P and X	*Lactobacillus curvatus*	Italian salami	pH 2–8 Temperatures range 4–121°C NaCl 2–10%. *L. monocytogenes.*	Barbosa et al., 2016
Nisin A (Nisaplin)		Milk pudding	Nisin A is able to control spore-forming bacteria and increase life span.	Oshima et al., 2014
Nisin A and Z	*Lactococcus lactis*	Dutch-cheese	Cell-free supernatant (CFS) from the bacteriocin synthesizing strains have low level of reduction capacity of *L. monocytogenes*	Dal Bello et al., 2012
Nisin Z	*Lactococcus lactis* W8	whole-fat skim and milk	Able to commute 5 log CFU/ml of *L. monocytogenes* to subtle levels after 16 h incubation at 8°C in skim and fat milk	Mitra et al., 2011
Pediocin SA-1	*Pediococcusacidilactici*	Dry sausage	Spoilage of Pathogens pH 3–12 100°C to 121°C for 60 min	Papagianni and Anastasiadou, 2009
Plantaricin	*Lactobacillus plantarum* LP 31	Fermented sausage in dry condition	Low pH with High temperature	Müller et al., 2009

One of the distinct roles that bacteriocin plays is in ecological homeostasis, where they help to maintain dynamics within a population within a particular species.

## Nisin

World Health Organization (WHO) and US Food and Drug Administration (FDA) gave the approval that the bacteriocin Nisin can be applied in food factories (Delves-Broughton, 2005). Nisin was first identified in 1928 from fermented milk cultures and was treated as a biopreservative in England in 1953 due to its ability to inhibit microbes, which is considered beneficial for the preservation of food (Costa et al., 2019). Nisin is 34 amino acids long with a MW of 3,354 kDa bacteriocin. Gram-positive bacteria mainly, *Lactococcus* and *Streptococcus*, strains typically produce Nisin. Nisin, a phase I bacteriocin (lantibiotic) containing five rings of lanthionine is soluble in water and its solubility increases by decreasing pH value, with a high melting point of 57 mg/mL at pH 2–3. (Abts et al., 2011). Nisin is considered safe for human beings at a concentration lesser than 83.25 mg/kg and lesser than 66.7 mg/kg for mice. Nonetheless, at a concentration of 300–400 mg mL^–1^ it might cause a preventative impact on humans (Kitagawa et al., 2019). Nisin has its inhibitory actions against Gram-positive foodborne microbes, like *Bacillus cereus*, *Clostridium botulinum, Listeria monocytogenes*, and *Staphylococcus aureus* (Zhao et al., 2016). It was studied that the synergistic effect of nisin consisting of antibiotics is effective to inhibit or kill the development of gram-negative microorganisms. There are several bio-engineered and natural variations of nisin which are delineated. Natural nisin is extricated from *Lactococcus* or *Streptococcus* strains through the absence of any gene modification, whereas *Lactococcus* and *Streptococcus* strains are able to produce bio-engineered Nisin through gene modification to improve its inhibitory impacts against pathogens which are gram-negative (Piper et al., 2011). For example, nisin Z is naturally synthesized by *L. lactis NZ22186*, but nisin Z N20K is extricated through gene modification of *L. lactis NZ9800* (Piper et al., 2011). Nisin is considered to be effective in preventing spoilage of food as it is able inhibit numerous food-borne microorganisms in a various range of food varieties, else in fluid or solid form (Choyam et al., 2019). Nisin is mostly used in cheese to increase its shelf life by inhibiting the development or to kill *L. monocytogenes* and *S. aureus* (Silva et al., 2018). Previously it was reported that the addition of nisin to the food lattice enables the prevention of milk spoilage by inhibiting the development of *Bacillus cereus*, *Clostridium botulinum*, and *Clostridium perfringens* (Gharsallaoui et al., 2016). Furthermore, *C.botulinum, B. cereus, and L. monocytogenes* are found in culinary, dairy, pastry shop items, and refreshments, leading to food spoilage and bacterial disease. Nisin is able to prevent the development of these microbes and delay the expiration date (Smith et al., 2004). *C. botulinum* and *L. monocytogenes* are usually found in meat items which can be inhibited by Nisin (Scott and Taylor, 1981).

## Enterocin

Enterocin is a round-shaped bacteriocin with 70 amino acids chain, produced by *Enterococcus spp.* Gram-positive bacteria are classified as LAB (Grande Burgos et al., 2014). Two types of Enterococcus, namely *Enterococcus faecalis* and *Enterococcus faecium* are found in the human digestive organs (Dubin and Pamer, 2014). Enterocin is classified into four groups while classes II and III enterocins, for example, enterocin AS-48, grab the attention due to their inhibitory ability against a broad spectrum of microbes causing food spoilage. Basically, the use of enterocin in food preservation is divided into two forms, either *in situ* production where enterocin-inducing species are directly administered to food to produce grist enterocin, or sometimes refined or slightly purified enterocin are added to the diet to prevent decay (Khan et al., 2010). The *Enterococcus species* are utilized shrewdly as it is a high-quality starter culture in the dairy food industry manufacture, especially cheeses (Terzić-Vidojević et al., 2015). Several enterococcal strains synthesize enterocin that can restrain the development of some other foodborne microbes in food by extending the storage time (Khan et al., 2010). However, numerous scientists have demonstrated that the utilization of purified or semi-purified enterocin for food preservation is found to be extra advantageous compared to *in situ* enterocin synthesis because of a few adverse consequences of enterocin-synthesizing strains to the hostile environment of the food (Silva et al., 2018).

Nowadays, the practice of having vegetables ready to be eaten with or without food preparation indicates malnutrition. In any case, the consumption of ready-to-eat vegetables can lead to foodborne contamination, causing diseases in humans (Luna-Guevara et al., 2019). To resolve this crisis, *E. faecalis* A-48-32-synthesized enterocin, namely AS-48, is a productive antimicrobial that inhibits the development of *Paenibacillus spp*., *S. Aureus, B. cereus*, and *Bacillus macroides* in fresh vegetable sources. A total of 10 mg mL^–1^ of enterocin AS-48 is found to be sufficient against the development of *B. Macroides* and, *B. cereus*, *S. aureus*, *Paenibacilluspolymyxa*, and *Paenibacillusamylolyticus* in food (Grande Burgos et al., 2014). Apart from this, enterocin AS-48 has been used for preserving sprouts, canned fruits, and soybeans due its ability to restrain the development of *L. monocytogenes* with a MIC value of 1 mg/mL and *Bacillus coagulans* with a MIC value of 6 mg/mL (Grande Burgos et al., 2014). The dairy product milk is rich in protein and is consumed by people and broadly utilized in the production of cheese. The high nutrient content of this product exhibits high chances of contamination. Enterocins are considered to be effective for preserving milk items. Several studies have been conducted to determine the ramifications of enterocin inhibitors to the bacterial species present in milk-based products (Gupta et al., 2016; Sharma et al., 2020). Enterocin A and Enterocin 416K1 isolated from *E. faecium* CTC492 and *E. casseli*-flavus IM 416K1, respectively, are applied in cottage cheese to prevent the development of *L. monocytogenes* with a MIC value of 4.57 mg/mL (Vimont et al., 2017).

Enterocins A and Enterocins B have been used jointly for natural preservation of munster cheddar against *L. monocytogenes* (Aspri et al., 2017). *E. faecium F58*-mediated Enterocins L50A, and B and *E. faecium* CRL35-mediated enterocin CRL 35 are used to preserve goat’s milk by preventing the development of *L. monocytogene*s (Khan et al., 2010). Enterocin AS-48 from *E. faecalis A-48-32* is able to suppress the development *B. cereus* to produce solid fat-free cheese (Grande Burgos et al., 2014). Several enterocins such as Enterocins A and Enterocins B, and sakacin K isolated from *E. faecium* CTC492 and *L. sakei* CTC494, respectively, are able to prevent the development of *Lactobacillus sakei* CTC746 to maintain the quality of cooked pork (Aymerich et al., 2002). Enterocins A and Enterocins B are produced in *E. faecium* CTC492 and have anti-bacterial effects against *Listeria innocua, L. monocytogenes*, and the microbes present in boiled sausages and dried cooked meat. It became clear that enterocin EJ97 from *E. faecalis* EJ97 works to improve vegetable puree (Zucchini) by preventing *B. macroides* and *Bacillus maroccanus* (García et al., 2004).

## Pediocin

A phase II bacteriocin, Pediocin, with a hydrophilic N-terminal and a hydrophobic C-terminal variable and molecular weight 2.7–17 kDa, was first sequestered in 1990 (Papagianni and Anastasiadou, 2009). It comprises 44 non-post-translationally amended peptides containing aromatic and aliphatic amino acids (Martin-Visscher et al., 2011). Pediocin, which is obtained from *Pediococcus strains*, is sturdy and stable at broad ranging pH, temperature, and several proteolytic enzymes. Pediocin is able to prevent bacteria responsible for food spoilage, like *Clostridium perfringens* and *Listeria monocytogenes*, by intriguing amino acids from cytoplasmic phospholipid layer membrane of selected cells (Nieto-Lozano et al., 2002). Similar to Enterocin, there are two ways to use pediocin in the diet, either by *in situ* technique by injecting into the diet with *Enterococcus* or *Lactobacillus* and *Pediococcus* strains to control the growth of microorganisms through the direct administration of pediocin in the total concentration of food matrix. Moreover, the direct addition of pediocin to food has a few impediments, leading to the changes in the amphiphilic nature and solubility (Silva et al., 2018). Pediocin was introduced as an important component in controlling food and beverage damage. For example, pediocin PA-1 from *Pediococcus acidilactici* MCH14 was shown to employ antibodies opposed to *L. monocytogenes’* healthy and long shelf life of dried sausages and boiled meat products. In addition, pediocin PA-1 is used to preserve fish files by suppressing *L. monocytogenes* development (Ming et al., 1997). Pediocin PA-1 is also able to inhibit *Bacillus subtilis* and *B. licheniformis* (Egan et al., 2016). Pediocin PA-1 isolated from *E. faecium* NCIM 5423 or *Lactobacillus plantarum* Acr, which has an antimicrobial effect against *L. monocytogenes*, is used in producing fermented soy milk with a long shelf life (Hassanzadazar et al., 2014).

## Leucocin

Leucocin is a class IIa bacteriocin obtained from *Leuconostoc* spp. Leucocin A is obtained from *Leuconostocgelidum* UAL187 and has an antimicrobial impact on developing *L. monocytogenes* in the protection of fresh meat, sausage, and milk products (Drider et al., 2006). A plasmid-intervened bacteriocin, Leucocin A has a molecular weight of 3.93 kDa with 37 amino acid residues (Balay et al., 2017). It has been resistant toward *L. monocytogenes* FSL C1-056, FSL J1-177, FSL N3-013, FSL N1-227, and FSL R2-499 with MIC of more than 2200.0 μM (Balay et al., 2018). Apart from this, Leucocin A has an inhibitory impact against *C. divergens* UAL9, which is responsible for meat spoilage with a MIC of 1.7 μM (Liu et al., 2011). Leucocin K7 is isolated from *Leuconostoc mesenteries* K7 is used in milk preservation due its inhibitory action as opposed to *L. monocytogenes*, which has a MIC of 28 μg/mL (Fu et al., 2018). Moreover, it was found that *Leuconostoeudomesenteroides* KM432Bz synthesizes leucocin B-KM432Bz that has an inhibitory effect along with a MIC of 64 nM on *L. monocytogenes* CIP 82.110, which have been found in dry Spanish style sausages (Tirloni et al., 2019).

## Other Types of Bacteriocins

Several other bacteria such as carnocyclin A, carnobacteriocin BM, leucocin A, leucocin B, lactococcinBZ, aureocin A70, sukacin, piscicolin 126, mycocin, natamycin, bacteriocin CAMT2, bacteriocin 7293A, and bacteriocin 7293B haves enhanced the shelf-life of food (Costa et al., 2019). The combination of *Carnobacterium maltaromaticum* UAL307 mediated carnocyclin A, carnobacteriocin BM1, and piscicolin 126 have an anti-bacterial effect against foodborne pathogens, including *E. coli* DH5a, *Pseudomonas aeruginosa* ATCC 14207, and *Salmonella* ATCC2 (Martin-Visscher et al., 2011). It was noted that *L. sakei*-produced sukacin is used to prevent *L. monocytogenes* development to store organic matter (Saraoui et al., 2018). Lactococcin BZ is used in the manufacturing of full-fat and skimmed milk as it exhibits antimicrobial impact against *L. monocytogenes* to prevent milk spoilage (Bizani et al., 2008). Aureocin A70 from *S. aureus* evince the blocking effect against *L. monocytogenes* in melted milk. The mold and yeast contamination in cheese, new dairy items, beverages, and processed meat are considerable fret in food industries as they acts on food wastage and the economical condition of the industry. Natamycin extracted from *Streptomyces gilvosporeus*is or *Streptomyces natalensis* are used in the food industry to solve this problem. It asserts to have antimicrobial impacts against molds and yeasts by increasing the shelf life of cheese, meat, beverages, and new dairy items (Elsayed et al., 2019). In addition, bacteriocins 7293A and 7293B from LAB and Weisselahellenica BCC 7239 were active antibodies to prevent Aeromonashydrophila, *E. coli, P. aeruginosa*, and *S. typhimurium* in meat and meat products (Costa et al., 2019).

## Bacteriocin-Mediated Food Preservation

To increase the shelf life, antibiotics or food preservatives like sulfur dioxide and nitrite are incorporated into the food source to arrest microbial development and possible contamination. But, most commercially used preservation methods are usually produced through long-term utilization of synthetic preservatives and chemical synthesis unfavorably affects the human body. Involvement of antibiotics in food items seems to be unethical. The bacteriocins synthesized by both or Gram-negative or Gram-positive bacteria alone are gene-encoded peptides that act as normal food preservative items. As a result, bacteriocins are found to be sensitive to some proteases whereas safe bacteriocin may be digested, therefore, non-functional bacteriocin-loaded small peptide food particles easily get digested in the human gastrointestinal tract (Cleveland et al., 2001). Bacteriocins are considered fundamentally safe as food preservatives. They are used as natural food preservatives in many types of food including cheeses, Portuguese matured meat, and yogurts (Yang et al., 2012). Among the food automation, nisin is an initial peptidyl antibacteria usually produced by *Lactococcus lactis*. It is a remunerative bacteriocin used as a food additive against food spoilage pathogens. It is the main food diversification in the U.S. Food and Drug Administration (USFDA) where it is authorized as a method of food preservatives in more than 45 countries. The other commercial bacteriocin is pediocin PA-1, retailed as Alta^®^2341, which prevents the proliferation of *L. monocytogenes* in meat items. Furthermore, bacteriocins like nisin are utilized in reamed potatoes, kimchi, or fermented cabbage, and new cut items as food additives. Enterocin AS-48 is utilized for preserving cider, canned vegetables, fruit and vegetable juices to reduce contamination. Also, Enterocin EJ97 and CCM4231 are utilized in zucchini puree and soy milk for reducing contamination, respectively (Settanni and Corsetti, 2008).

## Dairy Products

In addition to new developments in bacteriocin research on food use, utilization of refined bacteriocin in the dairy industry had its limitations. Often the sole use of bacteriocin is not able to provide adequate patronage against the contamination of dairy products. Also, the cost-effectiveness of the isolation followed by purification of bacteriocin is another drawback for commercial research of novel alternative bacteriocin. Furthermore, limited dietary control by health regulatory authorities like FDA and EFSA also bestow limitations on the consent of new bacteriocins as dietary supplements, and consequently, nisin and pediocin are only two bacteriocins now accessible for trade. The application of bacteriocin-producing bacteria to diminish bacterial contamination is an option for the use of purged bacteriocin as food preservatives. Many LAB species have an antiquity of secure use and are approved by the state of GRAS and QPS. Thus, the addition of these tiny insects to food sources provides a possible solution to control bacterial contamination. In addition, LAB is often used as the first culture in food fermentation. Therefore, scientists have investigated the production of bacteriocins through *in situ* technique *via* the addition of protective mechanisms that may produce bacteriocins during the production and storage of dairy foods.

Numerous studies have pivoted to the selection and the development of bacteriocinogen culture such as cell lysis-initiating agents to improve the ripening of cheese and flavor (Beshkova and Frengova, 2012). Additionally, bacteriocin-producing LAB has been used to interfere with the slow blasting of cheese. The recent cracking of the bug is an important cause of contamination of ripe cheese, which leads to the presence of consistency and spoilage of flavor, due to the full presence of *Clostridium* grains. Well-known methods of reducing *Clostridium* grains are often insufficient to prevent late cheese breakage and the use of bacteriocinogenic LAB materializes an alternative strategy. Among the different species/species of LAB that produce bacteriocin, *Lactococcus* sp. found some intrigue in the natural preservation of dairy foods.

The LAB varieties that produce Bacteriocin have been tested to improve the ripening and flavor of the cheese. These LAB cultures may result in limited modification of primary LAB and non-starter LAB (NSLAB) and intracellular release of proteins and peptidases, resulting in faster proteinololarization and cheddar maturation. Lc. Lactis-induced lacticin 3,147 accelerates the ripening of the cheese and averts premature spraying of cheese through the inhibition of Clostridia (Carmen Martínez-Cuesta et al., 2010). Different bacteriocin-producing strains have a lytic effect on early cultures. A bacteriocin-producing *Lactobacillus lactis* ssp. cremoris sustain the production of cheddar cheese and shoot up the level of starter lysis. Cheese made with bacteriocinogenic lead showed the increase in the lysis of cells and a higher concentration of free amino acids, as well as higher sensory testing points (Morgan et al., 1997).

Another launcher that produces bacteriocin Lc. lactis (maker of lacticin 3147) has been shown to control the propagation of unwanted microorganisms during cheese making. Cheese thru lacticin 3,147 primers showed low levels of NSLAB that remained unchanged for more than a year. A few types of lacticin 481-producing Lc. Lactis was also verified for a decrease in the number of developing lactococcal cultures in cheese production which continued to grow at a low rate. As a rapid consequence of starter lysis and the corresponding enzyme release in the cheddar matrix, which can increase ripening of cheese. In addition, the cheese was tested through a three-strain starter system, which involves the production of bacteriocin which causes the conversion of the second type and the third type, which is resistant to the action of the bacteriocin, creating acid during the cheese production. Experimental cheeses produced with the three-phase starter system has showed a hike in lysis and a decrease in salt compared to cheese made without bacteriocin production (Morgan et al., 2002).

## Bacteriocin in Antimicrobial Film Coating

One of the methods for food preservation, especially for raw and uncooked food, is by applying edible films or coating the food with antimicrobial substances. Application of this method, with bacteriocin, ensures that the food products are under the control of pathogenic microorganisms. The coatings or films of antimicrobial substances are usually made up of biopolymers layer which are generally thin, which help in the modification of the surrounding food atmosphere and also acts as a barrier between the environment and the food by increasing the safety and the quality and the functionality of the foods without causing any change in their nutritional properties. Bacteriocins which are purified or bacteriocin obtained from bacteria were used in the packaging and storing system as they are effective in resisting the growth of pathogenic microorganisms. Hydrocolloids are mainly used in cheese production. These hydrocolloids are mainly biopolymers that are applied as a coating or as films. Bacteriocins are used in dairy food production as they boost stability, make the food safer, and also increase the shelf life of the food.

**FIGURE 4:**
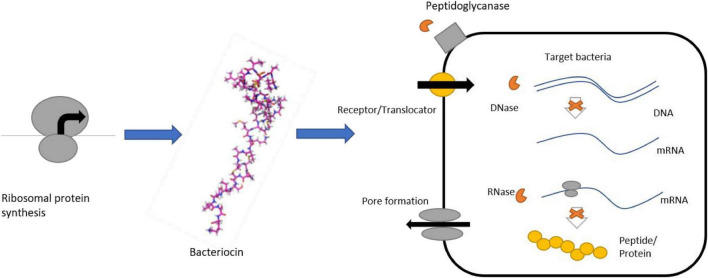
Bacteriocin production and its antibacterial activity.

Effective research on the synthesis of purified bacteriocin in edible pores provides a limited reduction in L-like bacteria *monocytogenes*. Cheese, especially young cheeses, are short-lived due to the high content of casein, lipids, and water present in them. Due to the complexity of cheese amalgamation and production, it, therefore, supports the development of microorganisms that cause decay and degradation which increases the risk of foodborne infections and reduces the quality and suitability of cheese (Ramos et al., 2012). Another problem is the post-process contamination, which can be overcome through the use of edible clothing and films mixed with bacteriocins that improve safety and increase the shelf life of the cheese. Studies have shown the use of a non-cellular film (CFS), which contains substances such as the bacteriocin of *Lactobacillus curvatus* P99, to control L. *monocytogenes* development in cheese ‘Prato’ sliced. These films contain a bactericidal compound of CFS that can restrain *L. monocytogenes* in a 10-day dose in 4°C (Marques et al., 2017) (Figure 4).

## Metabolomics

The field of metabolomics is a fundamental part within the omic science and can be used effectively in studying various aspects of food and associated metabolism. The mechanism of systemic identification along with quantification of metabolites is termed as metabolomics (Idle and Gonzalez, 2007). The small molecules that are produced during the time of metabolism can be fetched for understanding the metabolic pathway (Liu and Locasale, 2017). The compounds possessing a size below 1,500 Da make the process of metabolomics analysis difficult. The matrices of food comprise various types of metabolites including carbohydrates, proteins, carotenoids, alkaloids, steroids, alkaloids, and various other types of volatile compounds. Thus the metabolome analysis of various types of biological systems and dairy products can be achieved by the use of various types of analytical approaches (Castro-Puyana et al., 2017). The metabolomics analysis comprises two important techniques comprising nuclear magnetic resonance (NMR), mass spectroscopy, and spectroscopy (Miggiels et al., 2019).

## Application of Omic Sciences in Food Safety

Synthetic biology is not considered under omic sciences, but this tool is effective in food safety. The use of various engineering designs in the application of food safety is termed as synthetic biology (Agapakis, 2014). The mechanism of synthetic biology can be used effectively in the process of designing an organism and the production of important biological compounds (Minami, 2013). Thus this acts as an important platform for the development of new compounds that can be used effectively as food (Masuda et al., 2012). This field of science has helped in the discovery of custom-designed organisms that can be used effectively for the purpose of food preservation. The deigning of organisms like *L. monocytogenes* and *S. enterica* can effectively express GFP and can be detected by the use of PCR analysis acting as important organisms to prevent false negatives during testing of the food samples (Murphy et al., 2007). The application of genomics with synthetic biology helps in the designing of new phages that can be used for the detection of pathogens and can also act as biocontrol (Awais et al., 2006; Schofield et al., 2011, 2013). The genetically engineered phages can be used effectively in the detection of pathogens like *E. coli* and *B. anthracis* (Awais et al., 2006; Schofield et al., 2011, 2013). Studies have shown that engineered phages possess the ability to detect pathogens easily from various matrices of food (Awais et al., 2006). The use of non-lytic bacteriophages in the biocontrol of *E. coli* is performed by the engineering of special type of proteins that is lethal for the host cell and kills *E. coli* effectively (Liu et al., 2012). The combinatorial approach of omics with synthetic biology will help in paving new dimensions that would prevent contamination and spoilage of food (Yu et al., 2008).

## Conclusion

An increase in the population has resulted in the demand for food safety and security. There has been an increase in multidrug resistant microbes has turned into a difficult problem whereas finding or producing an alternative antimicrobial agent is becoming significantly imperative. Various conventional detection techniques were not suitable to understand the microbial flora associated with food. Various recent technologies of Omic sciences has resulted in profound detection of organisms and other biomolecules that are associated with the food. The use of bacteriocins can be also an alternate strategy in mitigating such issues. Thus understanding the structure, chemical composition, and metabolomics of the organisms results in a better investigation and the use of proper therapeutics in killing the targeted organisms.

## Author Contributions

BD, SiP, DL, MN, TS, and RR conceived and designed the experiments. SiP, VU, DL, MN, SoP, TS, RR, RA, MA, and MK contributed to the writing—original draft preparation. SiP, VU, DL, MN, AA, SiP, TS, RR, and MK contributed to the formatting and editing according journal guidelines. SiP, VU, DL, MN, SiP, TS, RR, RA, and MK contributed to the writing—review and editing. All authors have read and agreed to the published version of the manuscript.

## Conflict of Interest

SiP was employed by NatNov Bioscience Private Ltd. The remaining authors declare that the research was conducted in the absence of any commercial or financial relationships that could be construed as a potential conflict of interest.

## Publisher’s Note

All claims expressed in this article are solely those of the authors and do not necessarily represent those of their affiliated organizations, or those of the publisher, the editors and the reviewers. Any product that may be evaluated in this article, or claim that may be made by its manufacturer, is not guaranteed or endorsed by the publisher.
